# Silica Hydride: A Separation Material Every Analyst Should Know About

**DOI:** 10.3390/molecules26247505

**Published:** 2021-12-11

**Authors:** Joseph J. Pesek, Maria T. Matyska

**Affiliations:** Department of Chemistry, San Jose State University, San Jose, CA 95192, USA; maria.matyska-pesek@sjsu.edu

**Keywords:** HPLC, aqueous normal-phase, dual retention modes, surface water layer

## Abstract

This review describes the development, special features and applications of silica hydride-based stationary phases for HPLC. The unique surface of this material is in contrast to ordinary, standard silica, which is the material most frequently used in modern HPLC stationary phases. The standard silica surface contains mainly silanol (Si-OH) groups, while the silica hydride surface is instead composed of silicon-hydrogen groups, which is much more stable, less reactive and delivers different chromatographic and chemical characteristics. Other aspects of this material are described for each of the different bonded moieties available commercially. Some applications for each of these column types are also presented as well as a generic model for method development on silica hydride-based stationary phases.

## 1. Background

It has been more than 30 years since the concept of fabricating a silica-based HPLC stationary phase having a surface composed of silicon-hydride (Si-H) moieties instead of silanols (Si-OH) was first formulated [1,2,3]. The original approach proposed has been modified extensively over the years so that a proprietary process is now used in a commercially available product [4]. A substantial number of research articles and reviews have been published over the years documenting the evolution of silica hydride-based stationary phases from a hypothesis to a proven experimental concept that can function in a manner different from existing HPLC materials [4]. This review will cover three different areas that are of importance to users of HPLC that can be used for column selection: a) the unique features of silica hydride-based phases that distinguish them from other commercially available materials, b) various applications by column type that can serve as illustrations of the usefulness of these materials, and c) typical steps used in the development of methods on silica hydride stationary phases. 

## 2. Salient Features of Silica Hydride Stationary Phases 

**Scheme 1:**
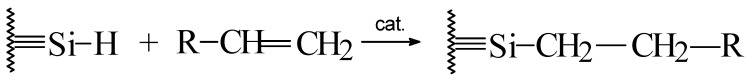
Hydrosilation reaction.

The bonding technology used to make these materials, hydrosilation, is completely different than that used for columns based on ordinary silica, organosilanization. The latter utilizes an organoilane reagent that reacts with the silanols on the surface to bond an organic moiety. Hydrosilation involves reacting an olefin or alkyne compound with the Si-H group to attach the organic species to the surface. The production of silica hydride results in approximately the same number of Si-H groups on the surface as ordinary silica has silanols. However, at the end of the bonding reactions, the remaining groups on the stationary phase are polar silanols for ordinary silica but nonpolar Si-H for the silica hydride phases. For silica hydride phases, the organic moiety is attached via a direct Si-C, while for ordinary silica, the linkage is Si-O-Si-C. The direct silicon-carbon bond provides much higher stability than the siloxane linkage obtained with organisilanization. In addition, the hydrosilation reactions have proven to be robust and reproducible (Scheme 1). For the same analysis on different lots of the same column material, variations in retention times or capacity factors (k) are no more than a few tenths of a percent RSD. 

There are numerous features that distinguish silica hydride from the ordinary silica used to fabricate the vast majority of commercially available HPLC stationary phases. The most obvious of these differences are the surface properties of these two materials. The surface of ordinary silica is populated by polar silanol groups (Si-OH) while the surface of silica hydride is composed of nonpolar silicon hydride moieties (Si-H). This profound difference has significant ramifications when comparing the two materials with respect to physical properties and chromatographic behavior. Because of the polar nature of silanols, many analytes can be adsorbed on the surface of stationary phases fabricated on ordinary silica. Even extensive endcapping leaves a significant number of silanols on the surface. However, silica hydride materials are composed of almost entirely Si-H groups that precludes the adsorption of most analytes on the surface. Another consequence of this difference in surfaces is that ordinary silica strongly adsorbs water leading to an aqueous layer of at least 4–10 monolayers in contrast to silica hydride having less than a monolayer of water under most chromatographic conditions [5,6]. This is especially significant when silica-based materials are used in the hydrophilic interaction liquid chromatography (HILIC) mode. Most HILIC analyses depend on a partitioning of analytes from the mobile phase to this adsorbed water layer as a means of chromatographic retention. Since such a layer does not exist on silica hydride materials, these phases utilize other mechanisms for polar compound retention (see discussion below). Another consequence of the lack of a water layer is the rapid equilibration between runs in gradient analysis or rapid equilibration when mobile phase conditions are changed for different analyses or method development. Another ramification of the absence of a significant water layer on the silica hydride surface is evident in organic normal phase chromatography. Since ordinary silica readily absorbs water, it must be carefully removed from the mobile phase; otherwise, the results can vary significantly from run-to-run on both an intraday and interday basis. However, the low affinity for water on a silica hydride surface eliminates the need to scrumptiously dry mobile phase solvents for organic normal phase chromatography. 

The retention for most nonpolar compounds on silica hydride columns is similar to that for ordinary silica-based stationary phases, i.e., hydrophobic interactions between the analyte and a bonded organic moiety such as C18 or C8. Thus, typical reversed-phase HPLC analyses can be done on these types of silica hydride phases. However, the mechanism of retention for polar compounds on silica hydride stationary phases was an issue for many years, since the surface is hydrophobic, yet strong retention of hydrophilic analytes was observed for a broad range of compounds in the aqueous normal phase mode. It was determined [7] that in mobile phases with a high content of an organic solvent such as acetonitrile, auto-dissociation of water occurs on silica hydride with hydroxyl ions prevalent on the surface of the material, giving it a negative overall charge. This phenomenon is similar to what happens to oil droplets in mixed solutions of organic solvent and water. Thus, retention of compounds having a positive charge or those having a positively polar component such as an amine group occurs by charge attraction and negatively charged or polarized molecules by displacement. 

**Figure 1:**
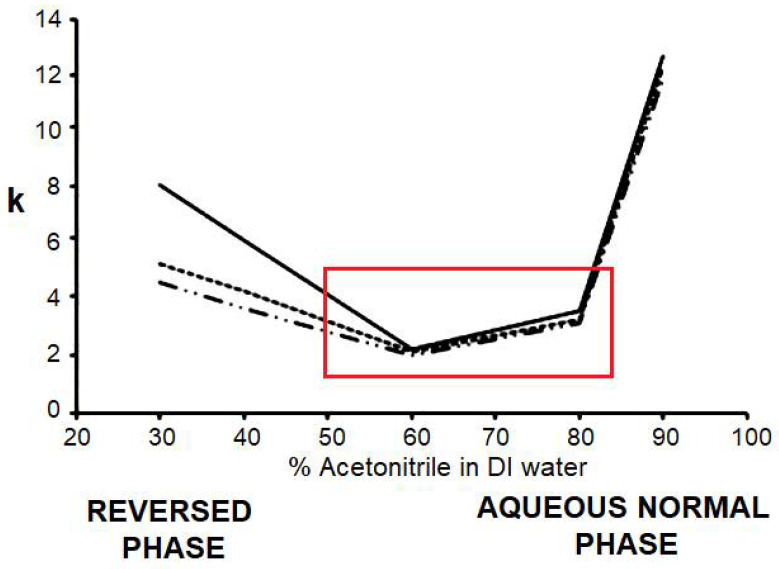
Retention map (k vs. % organic in mobile phase) for analytes on a silica hydride-based column.

The ability to retain polar and nonpolar compounds applies to all stationary phases created on a silica hydride surface. A plot of retention as a function of the percentage of organic component in the mobile phase results in a U-shaped curve (Figure 1). On the left-hand side of the graph, at low organic or high water content, reversed-phase retention is observed. On the right-hand side, at high organic or low water content, normal-phase retention is obtained. At intermediate mobile phase compositions, it is possible to have both reversed-phase and normal-phase retention operating simultaneously. For compounds with significant polar and nonpolar components in its structure, retention can be achieved in either mode, thus giving the analyst more options in developing a suitable method. This is a hypothetical graph, and the exact shape depends on both the analyte and the stationary phase. Other phases, such as certain fluorinated bonded compounds, also display this behavior to a limited extent in comparison to the broad range of retention properties exhibited by silica hydride-based materials. It has also been demonstrated that silica hydride can be prepared under supercritical fluid conditions but only in very small quantities [8].

## 3. Significant Applications by Column Type

While all silica hydride columns can operate in both the reversed-phase and normal-phase modes, the bonded moiety usually determines what retention properties will predominate. Thus, a nonpolar bonded moiety such as C18 will generally be used for reversed-phase applications, but it still has normal-phase properties in contrast to most other stationary phase with this type of modification. More polar bonded groups, such as diol or amide, would generally be used in normal-phase applications, but it still retains some reversed-phase properties. The following sections contain applications for each type of commercially available silica hydride column, which at this time happens to be produced by only one company. Columns types that are not available include chiral, size exclusion, anion-exchange, gel permeation, sub two-micron particle size and core shell. 

### 3.1. Diamond Hydride

This is the most widely used of the silica hydride-based columns. It was the first stationary phase to display capabilities for analyzing a broad range of polar compounds. Even today, it has features that either are not available or less versatile than most HILIC phases. The ability of equilibrate rapidly in normal-phase applications under a variety of mobile phase conditions (usually two to three column volumes) is in most cases vastly superior to HILIC columns. Another aspect of the Diamond Hydride column is its use of low buffer concentrations in the mobile phase. Typical additive concentrations are in the 5 to 15 mM range, while many HILIC applications can be significantly higher (30 to 100 mM is not unusual). This can be particularly important when using mass spectrometry for detection. High additive concentrations can leave deposits on the ion source, thus lowering sensitivity and requiring frequently cleaning. While acetonitrile is the most common organic solvent used in ANP analyses on the Diamond Hydride column, acetone [9] and methanol [10] have been shown to be applicable as well. Another feature of this stationary phase is its ability to retain certain strongly polar compounds in the normal-phase mode at very low concentrations of organic in the mobile. This often occurs at 20 to 30% organic content and has been referred to as “super ANP”. 

The analysis of polar compounds is an important component that occurs in a variety of different application areas, such as pharmaceuticals, metabolites, food, forensics, environment, and biotechnology. Many examples describing a variety of analyses of hydrophilic compounds can be found in the literature as well as on the Microsolv Technology website [11]. Some examples of the approximately 200 listed application notes through the website are the analysis of hydrophobic and hydrophilic peptides in a single run [12], benzodiazepines in urine [13], acrylamide [14], the pharmaceutical Xanax [15], anatoxin, which is a neurotoxin implicated in many poisoning incidents [16], and the common household product ingredient cetylpyridinium chloride [17]. Some examples of hydrophilic compounds analyzed on the Diamond Hydride in the literature are common metabolites [9], cathinones [18], collagen and elastin crosslinks [10], bactericidal targets [19], sugars [20], lipids [21], thiopurines [22], dietary supplements [23], juices and cereals [24], peptides [25], nucleotides [26], drug levels in human serum [27], and disease pathways [28,29,30]. In another report, the Diamond Hydride was combined with an RP column for a comprehensive survey of both polar and nonpolar metabolites [31]. One study compared the Diamond Hydride and three other Type-C stationary phases with respect to the relative retention of small molecules [32]. This range of applications displays the versatility of the Diamond Hydride column for polar compound analysis that goes beyond the capability of typical HILIC phases.

### 3.2. Phenyl

The phenyl phase having a hydrophobic moiety bonded to the silica hydride surface is generally used for reversed-phase applications, particularly those where the analytes contain aromatic or other types of unsaturated sites. Retention is enhanced for these types of analytes though π-π interactions with the bonded moiety.

**Figure 2:**
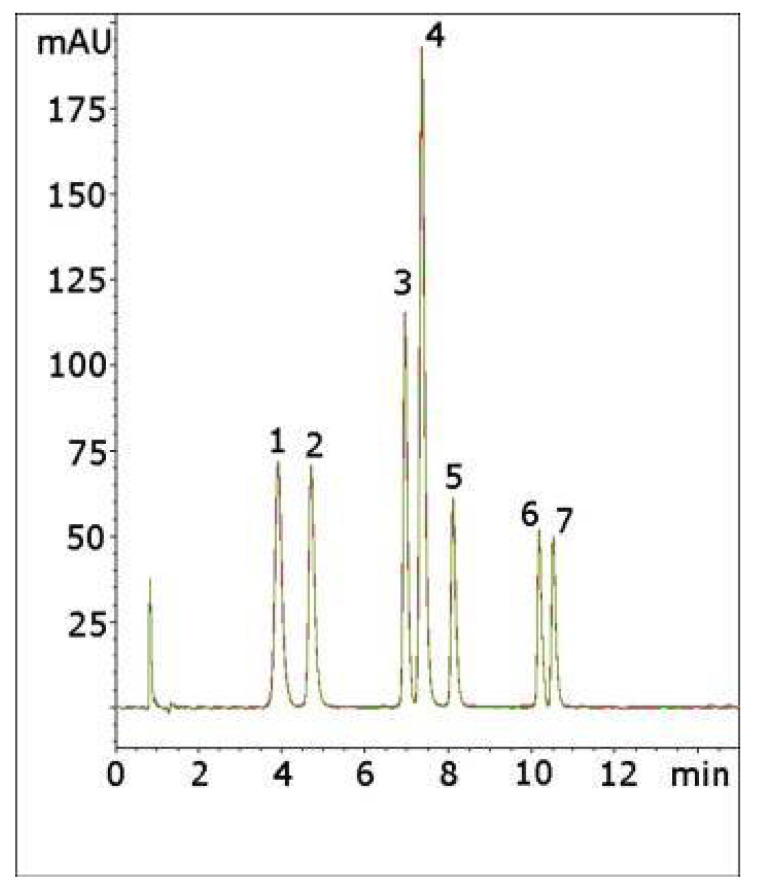
Gradient separation of common components found in cough syrup. Column, Cogent Phenyl Hydride 4.6 × 75 mm. Mobile phase, A DI water/0.1% TFA and B acetonitrile/0.1% TFA. Gradient: 0–2 min 5% B, 2–11 min to 50% B, 11–12 min to 5% B. Analytes: 1 = acetaminophen; 2 = pseudoephedrine; 3 = guafenesin; 4 = benzoic acid; 5 = methylparaben; 6 = dextromethorphan; and 7 = propyl paraben. Overlay of two column lots.

An example of an application for the phenyl column in the literature is the analysis of 16 common drugs of abuse by LCMS in under 8 min [33]. Under a different set of experimental conditions, the THC-delta-9-COOH can also be analyzed on the phenyl column. Additional articles citing the use of phenyl hydride columns involve the analysis of rice [34], mycotoxins in grains [35], and jaboticaba fruit [36]. However, there are more than 30 examples of analyses using the phenyl hydride column on the Microsolv website [37]. Included among the applications presented are the analysis of 10 phenolic acids in rice, methylenedioxymethamphetamine (MDMA) in plasma and the pharmaceutical compounds coricidin, fluoxetine, and ketorolac. An interesting example is shown in Figure 2 for the analysis of common components found in cough syrup [37]. This is a gradient analysis that is an overlay of five consecutive runs with an equilibration time of 3 min between runs. The run-to-run reproducibility of Cogent columns is one of their essential features, as well as rapid column equilibration between gradient runs.

### 3.3. Amide

A more recently developed silica hydride column is the amide stationary phase. The amide phase focuses primarily on hydrophilic molecules and is especially applicable to the analysis of sugars and various carbohydrates. Both a fundamental description of this phase and some examples of applications can be found in a published article [38].

**Figure 3:**
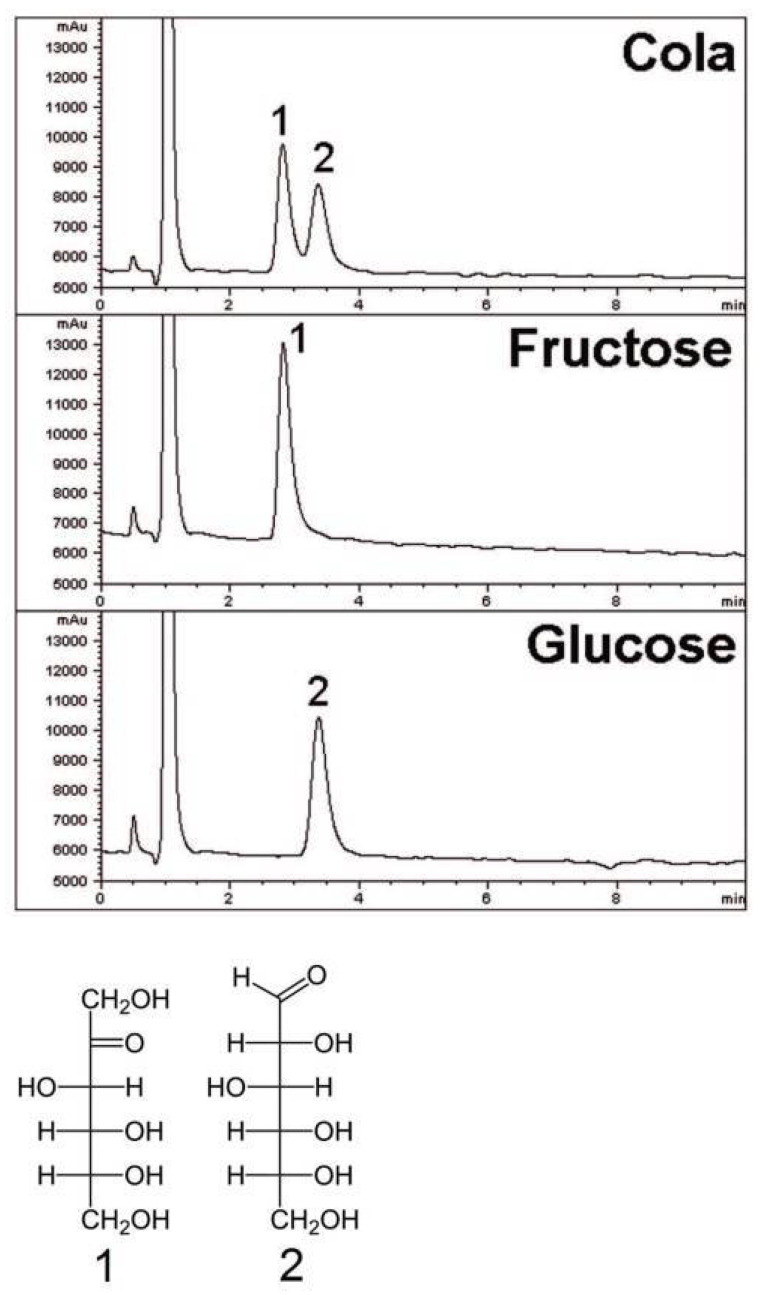
Separation of fructose and glucose on the silica hydride-based amide column. Column 4.6 × 50 mm. Mobile phase: 5% DI Water/95% acetonitrile.

Additional information on applications of the silica hydride-based amide column can be found online. A range of analyses are presented, relating to compounds that are not carbohydrates. An interesting example is the determination of the antidepressant fluoxetine (Prozac) in a capsule [39]. The compound is polar with a secondary amine, an ether linkage, and a trifluoromethyl group. The lot-to-lot reproducibility of this phase is also demonstrated in the application with three synthetic batches showing virtually identical retention times for the analyte. Nitrogen-containing compounds can often be difficult to analyze in reversed-phase due to adsorption on residual silanols and is often challenging in the HILIC mode. The separation of pyrilamine and 4-amino-3-chloropyridine with good peak shape demonstrates the ability of the silica hydride-based amide column for these types of compounds [39]. Another application involves the analysis of the pharmaceutical compound tizanidine in tablet form used to treat muscle spasms and cramps [39]. A particularly interesting application is shown Figure 3 for the separation of glucose and fructose in cola. These structurally similar compounds are a challenging separation, but can be done in the ANP mode under isocratic conditions using the amide silica hydride column [39].

### 3.4. UDC Cholesterol

This is a unique stationary phase that was first made with conventional bonding techniques on ordinary silica before it was subsequently adapted to a silica hydride matrix [40]. Cholesterol is a liquid crystal (solid with an ordered structure) in the native state. It was postulated and later proved that some of this ordered arrangement of the molecules exists even when one end is attached to a surface. In addition to hydrophobic interactions between the analyte and the bonded cholesterol moiety, there was discrimination based on the morphology of the stationary phase, which had a slot-like configuration. Thus, analytes that were more linear and could penetrate into the slots were retained better than bulkier molecules that were preferentially excluded from this restricted environment. The same exclusion phenomenon was also confirmed on the silica hydride cholesterol phase [41]. The phase was also tested for its effectiveness in small molecule separations [32].

**Figure 4:**
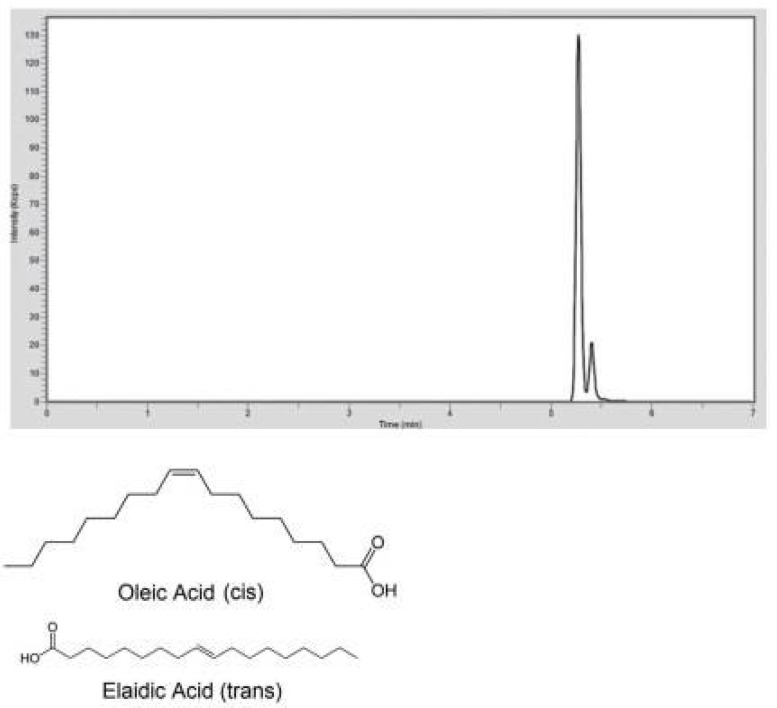
Separation of the isobaric C18 fatty acids on the cholesterol column. First peak is oleic acid (cis isomer) and second peak is elaidic acid (trans isomer).

Over the years, since its initial commercialization, a number of interesting applications have been developed. Among these are the separation of six serum corticosterones that can be used as disease markers in various clinical analyses [42], the drugs atropine [42] and doxcycline and methacycline [42], and the fruit juice component limonin [42]. A good example of the shape selectivity of the UDC cholesterol column is the separation of the monounsaturated C18 fatty acids shown in Figure 4 [42]. The two compounds, oleic acid (cis isomer) and elaidic acid (trans isomer), are separated on the UDC column with the cis isomer eluting first. This is due to the fact that the trans isomer is a linear molecule and penetrates deeper into the ordered stationary phase than the cis isomer, which is partially excluded due to its bent configuration.

### 3.5. UDA (Undeceanoic Acid)

While aqueous normal-phase chromatography is based on the presence of the silica hydride surface, the bonded moiety, as demonstrated with the amide phase, can also provide additional interactions that can facilitate the separation of hydrophilic compounds. In this case, the bonded moiety is a carboxcyclic acid compound. This functional group is the basis of weak cation-exchange properties for HPLC stationary phases. Thus, it can be surmised that this bonded phase would provide additional interactions for amines or other nitrogen containing compounds. An example of the effective use of this phase was demonstrated for the analysis of nucleotides related to clinical analysis [26]. Another interesting analysis successfully developed for the UDA phase was for the determination of ethyl glucuronide and ethyl sulfate [43]. These compounds are useful long-term markers for alcohol abuse and are often tested for in people in certain critical jobs, such as airline pilots or public safety personnel.

**Figure 5:**
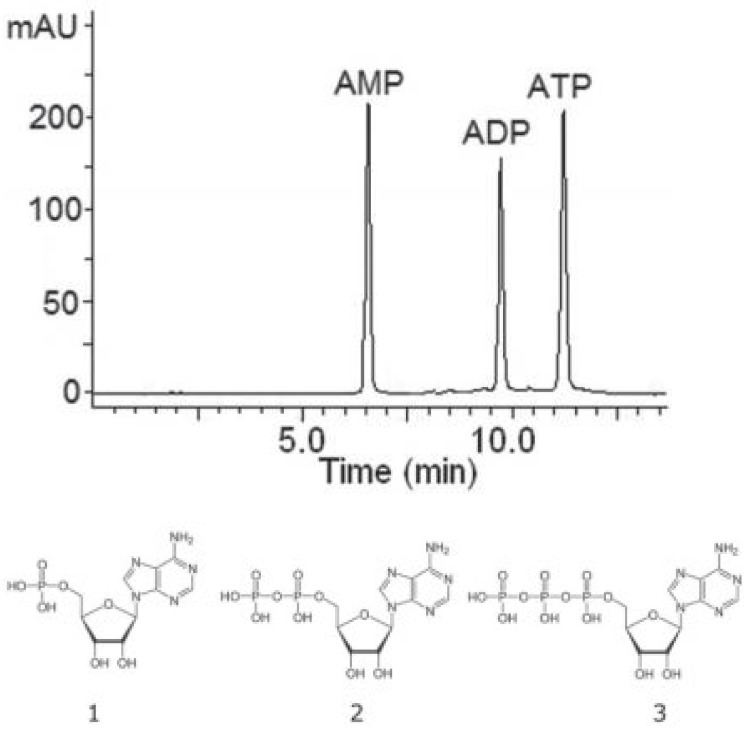
Separation of the three adenine nucleotides AMP, ADP and ATP on the Cogent UDA column. Peaks: 1. AMP—Adenosine 5′-monophosphate. 2. ADP—Adenosine 5′-diphosphate. 3. ATP—Adenosine 5′-triphosphate.

Additional examples of the use of this column can be found in online application notes. Pharmaceutical compounds containing nitrogen are another potential use of the UDA column. The antibiotic tobramycin was analyzed using a water/acetonitrile isocratic mobile phase with 0.5% formic acid [44]. Tablets containing vardenafil (Levitra) were extracted and then analyzed by HPLC using the UDA column [44]. A gradient method was developed in the ANP using a mobile phase containing DI water/acetonitrile with 0.1% formic acid. Excellent run-to-run repeatability was achieved with a 0.2% RSD for the retention time. A challenging analysis is the separation of the adenine nucleotides, AMP, ADP, and ATP, as shown in Figure 5 [44]. The chromatogram shown was obtained by gradient analysis in the ANP mode using a mobile phase A of water with ammonium formate buffer and mobile phase B of acetonitrile with ammonium acetate buffer. Excellent peak shape was obtained for these compounds, which often produce significant tailing on other types of stationary phases.

### 3.6. Bidentate C18

This stationary phase is the silica hydride version of the standard octadecyl column used in a large variety of reversed-phase applications. In addition to having a hydride surface, its other distinguishing feature is that it has a double attachment to the surface. Thus, the designation bidentate to denote this particular structural feature. As expected, the Bidentate C18 (BD C18) is amenable to a broad range of reversed-phase applications. Mycotoxins have a high toxicity and are a potential problem for human health. A number of these compounds have been analyzed in grain samples using the BD C18 column [35]. The nutritional supplement resveratrol in capsule form and in wine has been analyzed for both the *cis* and *trans* isomers [45]. Limonin, a bitter substance that can affect juice quality, has been analyzed on the BD C18 column [46]. Both reversed-phase and aqueous normal-phase performance was evaluated for a set test samples [32]. These are just some examples of analyses found in the literature for this stationary phase.

Online application notes provide additional examples of the usefulness of this column. A significant number of protocols involve pharmaceutical compounds. Among these are information for the separation of the isobaric opioid drugs morphine and hydromorphine [47], atorvastatin [47], the laxative bisacodyl [47], and the muscle relaxant dantrolene sodium [47].

### 3.7. Bidentate C8

While most reversed-phase applications are done with a C18 column, there are some instances when the analytes are so hydrophobic that these compounds take an unusually long time to elute or may not elute at all. Therefore, the way to reduce these interactions between the bonded moiety and the analyte is to make the stationary phase less hydrophobic. The simplest way to accomplish this is to shorten the bonded alkyl change. Hence, using a C8 (octyl) moiety accomplishes this goal. The C8 bonded phase on silica hydride has the same advantage as the C18 column with the ligand having a double attachment to the surface. An interesting application using the BD C8 column for the analysis of bisphenol A on carbonless paper often used as receipts for copying has been published [48]. Two extraction methods, one via digestion and another via migration, both gave satisfactory results with reproducible retention times.

**Figure 6:**
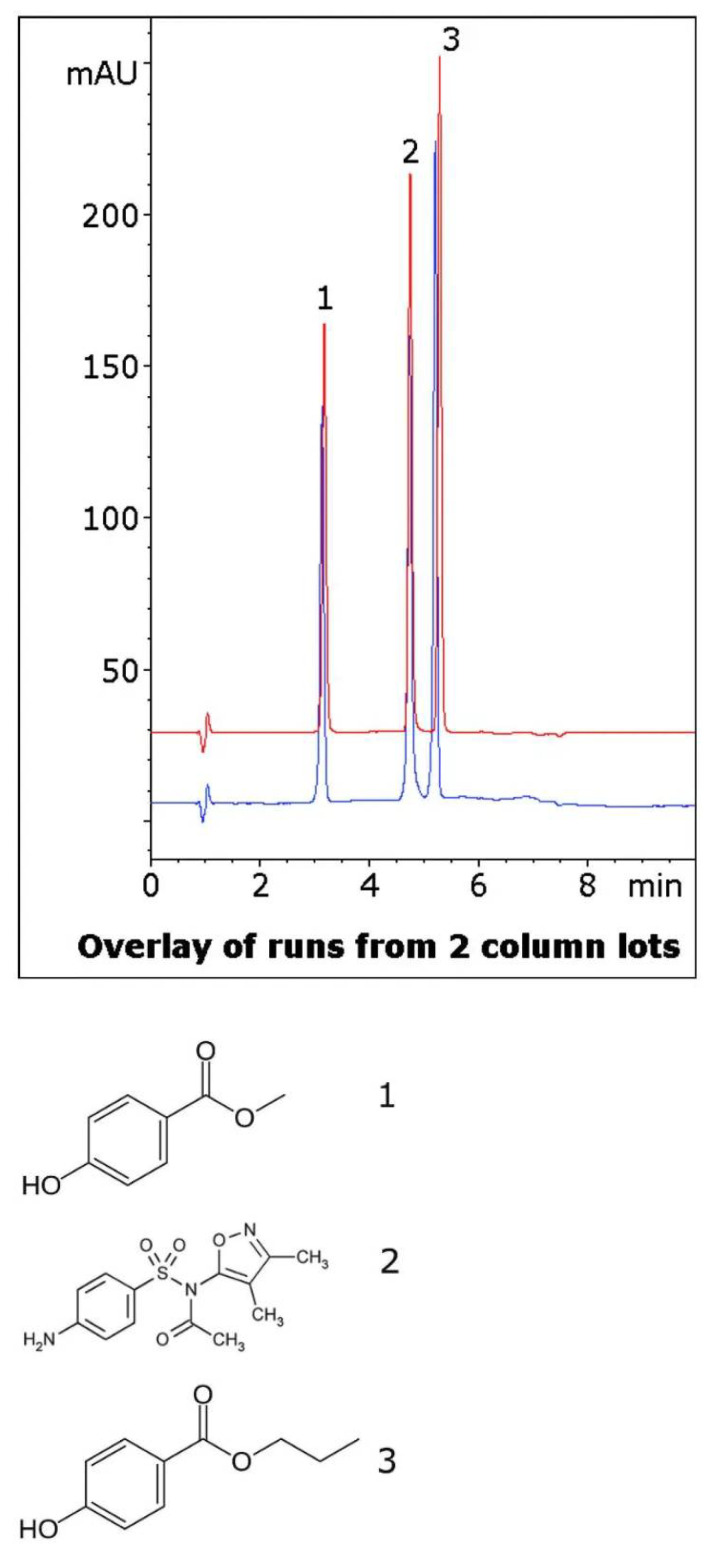
Separation of the drug sulfisoxazole acetyl and two preservative reference standards on the Bidentate C8 column. Peaks: 1 = methyl paraben; 2 = sulfisoxazole acetyl; 3 = propyl paraben.

There are numerous applications which can be viewed online for this phase. Many involve applications to pharmaceutical analysis. A protocol has been developed for the over-the-counter antihistamine chlorpheniramine maleate to identify the principal ingredient as well as a number of potential impurities [49]. Others include the antimalaria agent Clindamycin [49], the analgesic tramadol with data from two column lots demonstrating reproducibility [49], and prednisone, which is often used to treat arthritis or lupus, with the data showing excellent run-to-run reproducibility [49]. Another example of a pharmaceutical application is shown in Figure 6. This analysis involves the prodrug sulfisoxazole acetyl and two potential preservatives often used in the formulation [49]. The figure contains overlaid chromatograms from two different lots of material, demonstrating the reproducibility of the manufacturing process.

For very large molecules, particles with pore sizes around 110 Å are too small and may be excluded from interaction with the bonded organic moiety. Therefore, a silica hydride-based stationary phase with a bidentate-attached C8 group has been developed using a particle with a 300 Å pore size. A number of potential applications are documented to illustrate the usefulness of this particular stationary phase. The resolving power of this phase is demonstrated by the separation of two very similar cytochrome c proteins; one from horse heart and the other from bovine heart [50]. Another potential application is the separation of peptides [50]. This technique would be applicable to the characterization and purification of synthetic peptides. There is growing interest in the analysis of glycoproteins. It has been shown that the BD C8 phase can successfully retain these types of molecules with good peak shape [50].

### 3.8. Diol

The diol phase involves the attachment of polar organic moiety to the silica hydride surface. Therefore, it is generally used in the aqueous normal-phase mode for the analysis of hydrophilic compounds. Because the modified surface is different than the Diamond Hydride, the amide, and UDA phases, it has different selectivity and provides another option for trying to optimizing a particular type of separation. One study demonstrated how this column could be used to analyze a number of drugs of abuse [33]. Another investigation involved separating and analyzing uric acid cycle metabolites in the ANP mode where pre-column derivatization is not necessary as in many reversed-phase methods [51]. The diol phase was also included in a comparative study four different silica hydride phases [32].

**Figure 7:**
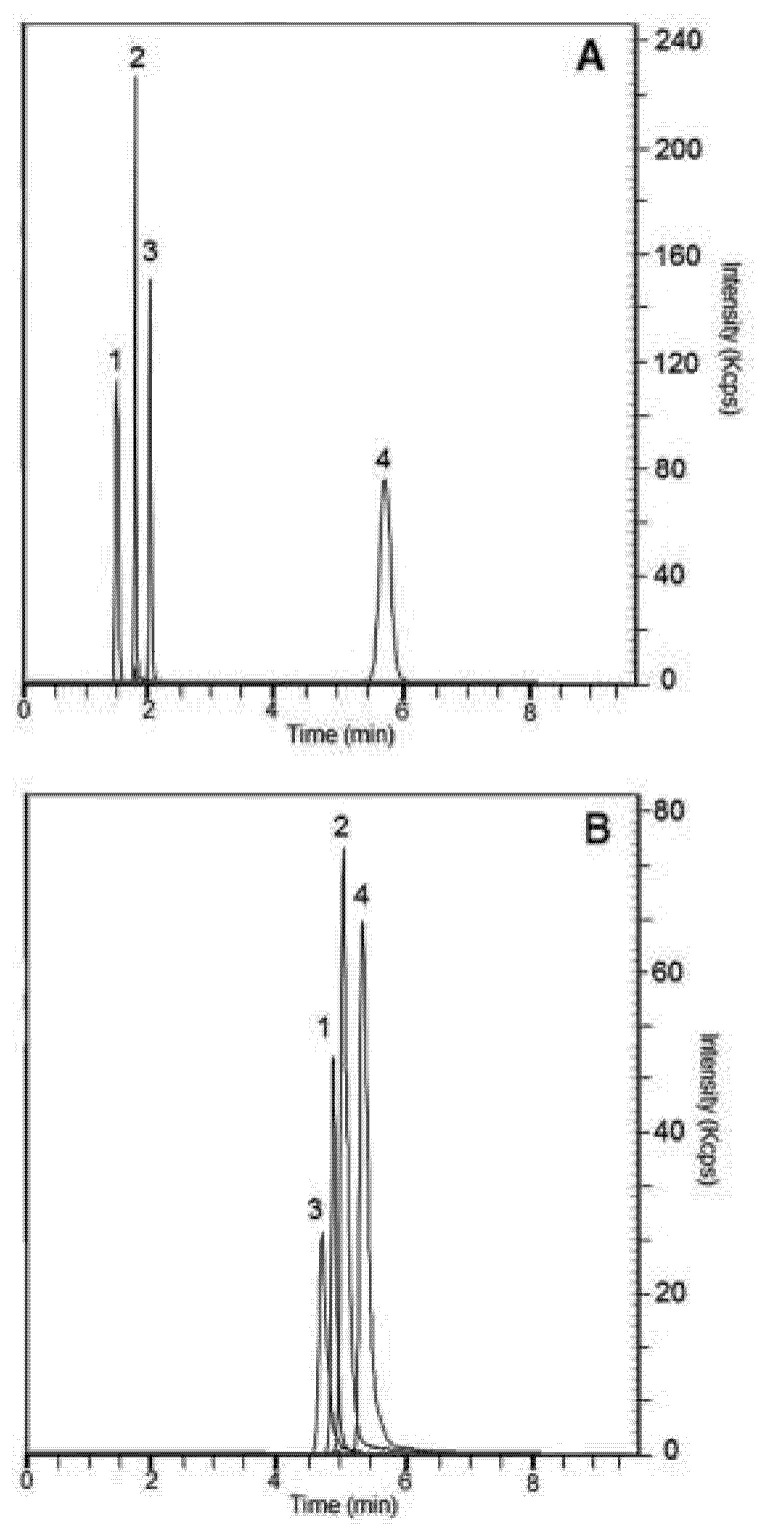
Separation of benzodiazepines in urine on the Cogent Diol column. (**A**) ANP mode and (**B**) RP mode. Peaks: 1. Temazepam 301.0739 *m/z* [M + H]+; 2. Diazepam 285.0790 [M + H]+; 3. Nordiazepam 271.0633 [M + H]+; 4. Midazolam 326.0855 [M + H]+.

Application notes for a number of other analyses are available that demonstrate some additional uses for this phase. The selectivity of this phase was highlighted in a study that separated ascorbic acid, niacin, riboflavin, folic acid, pyridoxine, metformin, and thiamine using a gradient ANP protocol [52]. In a similar type of experiment, the compounds warfarin, hydroxybupropion, and codeine in blood serum samples were analyzed with a simple liner ANP gradient. [52]. A good example of the dual retention capabilities of the silica hydride stationary phases is presented in separation of benzodiazepines on the diol column. The urine sample was analyzed with gradients in both the reversed-phase and aqueous normal-phase modes [52]. The chromatograms of both modes are shown in Figure 7.

### 3.9. Silica C

Silica C is the name used for an unmodified silica hydride particle. Because it has a low level of water adsorption (less than 0.5 of a monolayer), it should be an excellent choice for organic normal-phase chromatography. This feature is in direct contrast to ordinary silica, which strongly adsorbs water. Thus, when using ordinary silica in organic normal-phase chromatography, the mobile phase must the carefully dried to obtain reproducible retention times. Such precautions are not necessary for Silica C due to its low affinity for water. This concept was proven for the evaluation of phenolic compounds using traditional organic normal-phase chromatography on the Silica-C stationary phase [53]. Another example of an organic normal-phase application is the analysis of nonylphenol, a compound used in the synthesis of surfactants [54].

**Figure 8:**
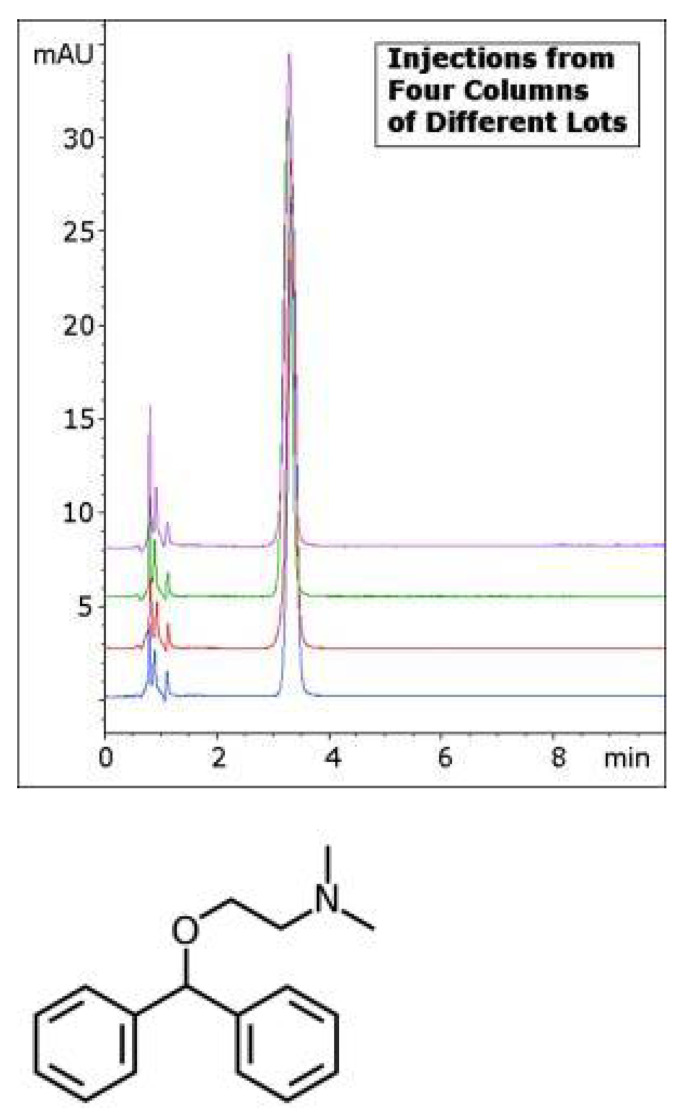
Analysis of diphenhydramine on the Cogent Silica-C column with runs on four different synthetic batches of stationary phase.

The Silica-C stationary phase has also been proven to be successful in the aqueous normal phase mode [55,56]. A number of pharmaceutical compounds have been analyzed using this stationary phase by ANP. A protocol was developed for the antihistamine diphenhydramine using an isocratic mobile phase of 50:50 water/acetonitrile with 5 mM ammonium acetate [57]. This particular example demonstrates the strong normal-phase retention properties of the silica hydride phases considering the large percentage of water in the mobile phase. In addition, this method was tested on four different lots of the Silica-C material, giving excellent reproducibility among columns (see Figure 8). Another drug, ketotifen, used for the treatment of asthma, was analyzed by a gradient ANP method [57]. This tertiary amine compound had strong retention on the Silica-C and the protocol developed gave excellent run-to run reproducibility. Phenylglycine used in the synthesis of lactam antibiotics was analyzed with an isocratic mobile phase consisting of 80:20 acetonitrile/water with 0.5% formic acid [57].

## 4. General Method Development Strategies on Silica Hydride Columns

It is important to note that while some principles of method development on ordinary silica-based columns are similar to those used for silica hydride stationary phases, there are some differences based on the surface properties of the two materials. In general, most reversed-phase strategies are reasonable similar for the two types of columns. However, there are often more significant differences when doing method development in aqueous normal-phase as opposed to HILIC. In any case, it is not always possible to make a direct transfer of a method developed on an ordinary silica-based stationary phase to a silica hydride column. Therefore, some method refinement may be needed to get optimum performance on the silica hydride stationary phase. Below are some general strategies that can be used for method development on the silica hydride-based stationary phases in each of the primary retention modes (reversed-phase and aqueous normal-phase) depending on the acid-base properties of the analyte.

### 4.1. Reversed-Phase

#### 4.1.1. Neutrals

Use a mobile phase consisting of either acetonitrile or methanol for the organic component and DI water. For most applications, the addition of 0.1% formic acid to both DI water and organic solvent is recommended, but it is essential when using mass spectrometry detection. If there are only a few components in the sample with not a large range of hydrophocities, then start with a high amount of organic (70–90%) in the mobile phase and increase the amount of water in 10% increments until the desired separation is achieved. For more complex samples (number of constituents or large hydrophobicity range), set up a linear gradient from 90% water to 10% water over 10 min. Adjust range of mobile phase composition and steepness of gradient (time) until a desired separation is achieved.

#### 4.1.2. Acids

It is essential to acidify the mobile phase using 0.05% phosphoric acid or 0.1% formic acid when analyzing acids in reversed-phase so that the target analyte will be neutral. Follow the same protocol above for neutrals when developing either an isocratic or gradient method.

#### 4.1.3. Bases

Many bases will have a substantial hydrophobic component and can be retained by the protocols described above. Some small bases are too polar to be retained in reversed-phase due to the positive charge on the amine group at low pH, and thus, it is better to utilize aqueous normal-phase. High pH is not recommended since it can damage the HPLC instrument and/or the stationary phase.

### 4.2. Aqueous Normal-Phase (ANP)

#### 4.2.1. Acids

To take advantage of the polar properties of acids, they must be ionized so a buffer of 10 mM ammonium formate or ammonium acetate at pH 6.5 is used as a starting point. Lower or higher molarity buffers can be used as well. For samples with few components, start at 50% water/10 mM buffer and increase the acetonitrile/10 mM buffer content in the mobile phase as needed to get the desired separation and retention. For more complex samples, use a test gradient starting at 90% acetonitrile/10 mM buffer and going to 20% over 10 min. Adjust as needed to get the desired resolution and retention.

#### 4.2.2. Neutrals

Only polar neutrals are likely to be retained in ANP. The same protocols for acids can be used for polar neutrals in ANP.

#### 4.2.3. Bases

To take advantage of the polar properties of bases, they must be ionized so a buffer of 0.1% formic acid or 0.2% acetic acid is used. Both isocratic and gradient protocols described above can be used for bases depending on whether the sample has a few components or is complex.

In general, it is best to follow the instructions provided with each column with respect to mobile phase solvents, pH, and mobile phase additives and buffers.

## 5. Conclusions

Silica hydride-based stationary phases are a unique material with respect to their chromatographic properties and separation capabilities. In many instances, they offer distinct advantages when trying to develop methods for the separation of challenging mixtures. In particular the analysis of polar compounds is facilitated by the unique surface properties of the silica hydride particles. In addition, the dual retention properties of silica hydride materials, for which all phases can be used in the reversed-phase as well as the normal-phase modes, offer new approaches for the separation of complex mixtures. The run-to-run, day-to-day, and column-to-column reproducibility of the silica hydride materials is excellent. The bonding of the organic moiety to the surface via a silicon-carbon bond provides durability to these materials. There exists in the scientific literature, as well as in online application notes, a broad array of analyses that document the versatility and benefits of using silica hydride-based stationary phases for HPLC. Because the materials are different than standard silica phases, moving from existing protocols on a conventional silica column to a silica hydride column may require method development. This is more likely when adapting an HILIC method to aqueous normal phase because of the significantly different retention mechanisms. Currently available phases do not provide chiral, size exclusion, anion-exchange, or gel permeation capabilities. The smallest particle size in use is 2.2 microns and core shell formats are not available.
